# Oncologic and Obstetric Outcomes Following Radical Abdominal Trachelectomy in Non-Low-Risk Early-Stage Cervical Cancers: A 10-Year Austrian Single-Center Experience

**DOI:** 10.3390/jpm14060611

**Published:** 2024-06-08

**Authors:** Melina Danisch, Marlene Kranawetter, Thomas Bartl, Magdalena Postl, Christoph Grimm, Eva Langthaler, Stephan Polterauer

**Affiliations:** 1 Department of Obstetrics and Gynecology, Comprehensive Cancer Center Vienna, Medical University of Vienna, Währinger Gürtel 18-20, 1090 Vienna, Austria; melina.danisch@meduniwien.ac.at (M.D.); marlene.kranawetter@meduniwien.ac.at (M.K.); magdalena.postl@meduniwien.ac.at (M.P.); christoph.grimm@meduniwien.ac.at (C.G.); stephan.polterauer@meduniwien.ac.at (S.P.); 2 Department of Pathology, Medical University of Vienna, Währinger Gürtel 18-20, 1090 Vienna, Austria; eva.langthaler@meduniwien.ac.at; 3 Karl Landsteiner Institute for General Gynecology and Experimental Gynecologic Oncology, 1090 Vienna, Austria

**Keywords:** cervical cancer, radical abdominal trachelectomy, fertility preservation, oncological outcomes, fertility outcomes

## Abstract

Radical trachelectomy allows for fertility preservation in patients with early cervical cancer not qualifying as “low-risk” as defined by ConCerv. This study reports on the 10-year surgical, oncological, and obstetrical experience of patients treated by radical abdominal trachelectomy at an Austrian tertiary care center. A retrospective chart analysis and telephone survey of all patients with FIGO stage IA2-IB2 (2018) cervical cancer treated by radical abdominal trachelectomy and pelvic lymphadenectomy between 2013 and 2022 were performed. Radical abdominal trachelectomy was attempted in 29 patients, of whom 3 patients underwent neoadjuvant chemotherapy. Three cases, including one after neoadjuvant therapy, required conversion to radical hysterectomy due to positive margins; four cases had positive lymph nodes following surgical staging and were referred to primary chemo-radiotherapy. Twenty-two (75.9%) successful abdominal radical trachelectomies preserving fertility were performed. According to final histopathology, 79.3% of tumors would not have met the “low-risk”-criteria. At a median follow-up of 64.5 (25.5–104.0) months, no recurrence was observed. Eight (36.4%) patients attempted to conceive, with a live birth rate of 62.5%. Radical abdominal trachelectomy appears oncologically safe in early-stage cervical cancers that do not fulfill the “low-risk”-criteria. Strict preoperative selection of patients who might qualify for more conservative surgical approaches is strongly recommended.

## 1. Introduction

In recent years, groundbreaking studies have paved the way for less radical surgical approaches in early-stage cervical cancers. While the “one-size-fits all” approach of a radical hysterectomy, including the removal of the uterus, cervix, upper vagina, parametria, and pelvic lymph nodes, is being increasingly questioned, the number of women expressing interest in fertility-preserving therapeutic options is ever-growing due to the increasing average child-bearing age in the Western world [1,2]. Moreover, fertility-sparing surgery was observed to gain implications for the psychosocial welfare of patients, who often experience feelings of depression and grief associated with loss of fertility [3].

Radical trachelectomy was first described by Dargent in 1994 using a vaginal approach and later in 1997, complemented by Smith using an abdominal approach [4,5]. Subsequently, the procedure has been implemented all over the world, demonstrating fertility preservation and oncologic safety [6,7]. Radical trachelectomy is a therapeutic option for women who are of reproductive age and still wish to conceive, diagnosed with early-stage squamous cell or adenocarcinoma, with limited endocervical extension and no evidence of lymph node metastasis on initial staging [8]. During the procedure, pelvic lymph nodes are typically removed, followed by the removal of the cervix, parametria, and the upper vagina. For early-stage cervical cancer, radical trachelectomy has equivalent oncologic outcomes to radical hysterectomy [9,10,11]. Also, obstetrical outcomes have been defined in sizable cohorts in recent years, reporting promising live birth rates at increased risk of preterm delivery [7].

Most recently, several studies substantiated evidence that more conservative surgical management in selected patients with “low-risk” early-stage cervical cancers appears oncologically safe. The ConCerv study defined tumors fulfilling the following criteria as “low-risk”: (1) FIGO 2009 stage IA2-IB1 cervical carcinoma; (2) squamous cell (any grade) or adenocarcinoma (grade 1 or 2 only) histology; (3) tumor size < 2 cm; (4) no lymphovascular space invasion; (5) depth of invasion < 10 mm; (6) negative imaging for metastatic disease; and (7) negative conization margins. Based on these criteria, both conizations followed by lymph node assessment (*n* = 44) in case of preferred fertility preservation, as well as simple hysterectomies followed by lymph node assessment (*n* = 40), appeared oncologically safe; positive lymph nodes were noted in 5% of cases, and three recurrences (3.5%) within 2 years were observed [12]. Similarly, Plante et al. reported simple vaginal trachelectomies in selected women with early-stage low-risk cervical cancer to be oncologically safe, applying comparable criteria as the ConCerv study [13]. Most recently, the SHAPE trial confirmed this hypothesis in patients with early cervical cancer without the wish to preserve fertility, demonstrating that pelvic recurrence rates following simple hysterectomies do not appear higher than following radical hysterectomies in tumors with lesions of <20 mm and with limited stromal invasion of <10 mm [14].

Following these findings, accurately selecting patients who wish to preserve fertility for either radical trachelectomy or more conservative approaches is of increasing clinical importance. As evidence of oncologic and obstetrical outcomes following radical abdominal trachelectomies in Germany and Austria is currently limited, and only few tertiary care centers in Central Europe perform the procedure, the present study aimed to retrospectively assess patients who underwent radical abdominal trachelectomies at the Department of Obstetrics and Gynaecology, Medical University of Vienna, Austria, over a timeframe of 10 years and contextualize outcomes and patient selection according to more recently proposed “low-risk” criteria [15].

## 2. Methods

The present study was designed as a single-center retrospective data analysis of all consecutive patients with early-stage cervical cancer, with International Federation of Gynecology and Obstetrics (FIGO) stage IA2-IB2 (2018) and the desire to preserve fertility, who underwent a radical trachelectomy at the Medical University of Vienna between January 2013 and December 2022.

Patients were selected as candidates for trachelectomy—and for evaluation in the present study—according to following criteria: (1) all premenopausal patients diagnosed with histologically confirmed early-stage cervical cancer, (2) who wished to preserve fertility and (3) for whom follow-up data at our institution was available; (4) only patients with stages IA2 to IB2 with tumors ≤40 mm irrespective of lymphovascular space involvement were given consideration for radical trachelectomy. Any involvement of the upper cervical canal with less than 10 mm tumor-free margin, as defined by pretherapeutic pelvic MRI, was considered a contraindication for upfront trachelectomy.

**Figure 1:**
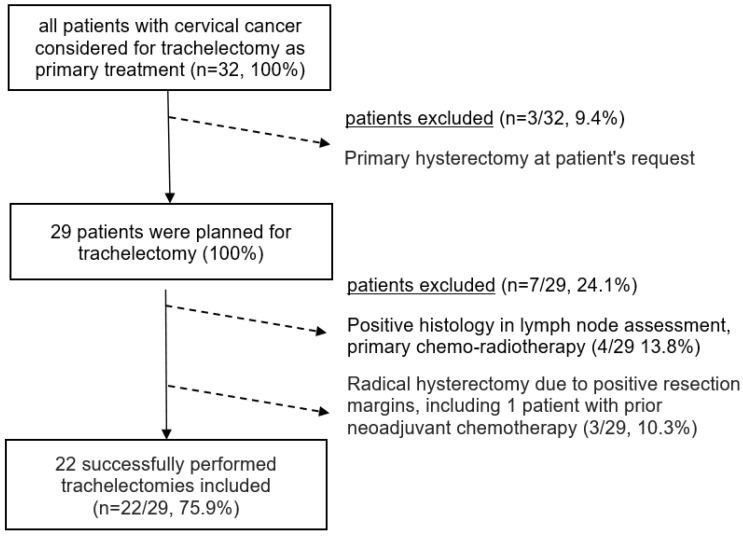
Consort diagram of all patients with cervical cancer considered for trachelectomy as primary treatment (*n* = 32).

(1) A patient age younger than 18 at the time of diagnosis, (2) primary surgical therapy in another hospital, or (3) reported active secondary malignancy within five years of diagnosis were defined as exclusion criteria; however, no patients had to be excluded from analyses; all patients undergoing trachelectomy within the specified timeframe were reported in the present study. Patient selection according to above-defined criteria is described in the consort diagram in Figure 1.

All patients considered for potential fertility-sparing management were reviewed by the department’s internal multidisciplinary tumor board prior to treatment initiation. Pretherapeutic staging included a clinical examination by a European Society of Gynaecological Oncology (ESGO) accredited gynecologic oncologist, a pelvic MRI with T2-weighted spin-echo sequences, and at least a thoracic CT scan, supplemented by full-body PET-CT, if available. All patients identified as potential candidates for fertility-sparing management were initially counseled on fertility-sparing approaches by the case-managing ESGO-accredited gynecologic oncologist. If patients desired further counseling or were recommended to undergo systemic treatment or radiotherapy that could affect fertility, they were referred to our department’s Division of Gynecological Endocrinology and Reproductive Medicine for specialized counseling by the “Oncofertility” Workgroup. All patients referred to radical trachelectomy underwent previous surgical pelvic lymph node staging; trachelectomy was only performed following negative results by either frozen section in the case of a one-staged intervention or by final histopathology in the case of a two-staged intervention. Until end-2016, routine management involved one-stage open systematic lymphadenectomy with intraoperative frozen section analysis; in the case of lymph node positivity as indicated by frozen section, the procedure was aborted. Starting end-2016, laparoscopic lymph node staging was routinely initiated. Since then, our institution implemented a one-staged blue dye—later replaced by indocyanine green (ICG)—based laparoscopic sentinel lymph node biopsy with intraoperative frozen section analysis, followed by radical abdominal trachelectomy via Pfannenstiel laparotomy in case frozen section yielded negative results. In the case of pathologic upstaging, the patient was referred to chemo-radiotherapy. Patients who underwent neoadjuvant chemotherapy followed by radical abdominal trachelectomy underwent pelvic laparoscopic lymph node staging before initiation of systemic therapy.

Radical abdominal trachelectomy was performed according to Querleu–Morrow-type C1, and the cervix, parametrium, and vaginal cuff were excised [16,17]. A non-resorbable cerclage was positioned, and the residuum of the cervix was then sutured to the vagina. Intraoperative frozen section analysis of all pathologic specimens was performed; in case involvement of the upper cervical canal was detected, further tissue was removed if possible. If no tumor-free margin of at least 10 mm to the internal os could be achieved, the trachelectomy was aborted, and a radical abdominal hysterectomy, according to Querleu–Morrow-type C1, was performed. All patients underwent routine oncological follow-up at our institution, and patients lost to follow-up were actively contacted before statistical analysis. No patient was lost to follow-up.

Statistical analysis was performed using SPSS^®^ (IBM Corp. Released 2020. IBM SPSS Statistics for Windows, Version 27.0, Armonk, NY, USA: IBM Corp.) for Windows. Descriptive statistics were performed. Categorical variables were described using percentages, and medians with upper and lower quartiles were used to describe continuous variables. Institutional review board approval was obtained from the Ethics Committee of the Medical University of Vienna (1641/2018).

## 3. Results

### 3.1. Descriptive Characteristics

Patients’ baseline characteristics are depicted in Table 1. Of the 32 patients who were considered for radical abdominal trachelectomy within the observational timeframe, 3 patients finally opted out and preferred to undergo radical hysterectomy instead; of the 29 patients (100%) subsequently planned to undergo radical abdominal trachelectomy with surgical pelvic lymph node assessment, 22 (75.9%) finally underwent the procedure successfully (Figure 1).

No case of infiltration of the parametria or the parametrial lymph nodes was observed, and no case of frozen section failure following one-staged surgical lymph node stagings was recorded. In one case, final histopathology revealed a tumor-free margin of <10 mm as measured from the inner os of the cervical canal after initially successful trachelectomy. Following a close margin, as reported by frozen section, an intraoperative re-resection was attempted, which did not result in a total free margin of at least 10 mm in final histopathology. After a discussion of the final results, the patient opted to undergo a hysterectomy, which was performed without complications and did not reveal any further residual tumor. Postoperatively, three patients experienced Clavien–Dindo grade III complications. Two patients required drainage of lymphatic cysts, and one patient required drainage of a pelvic abscess, which, however, resulted in full recovery without long-term symptoms.

At a median follow-up time of 64.5 (25.5–104.0) months, no disease recurrence occurred.

### 3.2. Neoadjuvant Management

Three neoadjuvant approaches were attempted by administering weekly carboplatin 2 mg/AUC and paclitaxel 60 mg/m^2^. Patients referred to neoadjuvant chemotherapy all had squamous tumors measuring 34 mm, 40 mm, and 40 mm, as determined by MRI during primary staging; in all cases, an involvement of the upper cervical canal with less than 10 mm tumor-free margin was suspected. After extensive discussion of staging results and patients insisting on fertility-sparing management, neoadjuvant approaches were offered, resulting in one partial response and two pathologically complete responses [18]. In the patient who experienced the partial response, the trachelectomy had to be aborted due to close tumor margins in frozen section, and the intervention was converted to a radical abdominal hysterectomy instead.

### 3.3. Obstetric Outcomes

Obstetric outcomes are depicted in Table 2. Only 8 (36.4%) out of the 22 patients who had a successful radical trachelectomy attempted to conceive. Reported reasons for patients not trying to conceive included fear of cancer recurrence and fear of preterm delivery. Five (62.5%) of the eight patients who tried to conceive subsequently became pregnant. Four patients conceived spontaneously, and one patient underwent successful in vitro fertilization and embryo transfer.

One patient (who later had a successful third-trimester delivery) experienced a first-trimester miscarriage. Four pregnancies were delivered by Caesarean section in the third trimester, three patients by scheduled Caesarean section at 38 weeks gestation, and one patient by emergency Caesarean section at 35 weeks gestation due to acute vaginal bleeding, the cause of which remained unclear. Of the four patients with live births, two patients experienced vaginal bleeding during the first trimester, in one case most likely caused by a decidual polyp. One patient experienced urinary retention in the second trimester, requiring an indwelling urinary catheter for a few days. Symptoms resolved at the end of the second trimester. No striking associations between clinical pregnancy rates and pretherapeutic FIGO stages were observed; of the four patients with successful third-trimester deliveries, one had FIGO IB1 and three an IB2 tumor. One patient with an IB2 tumor underwent pretherapeutic neoadjuvant chemotherapy.

**Figure 2:**
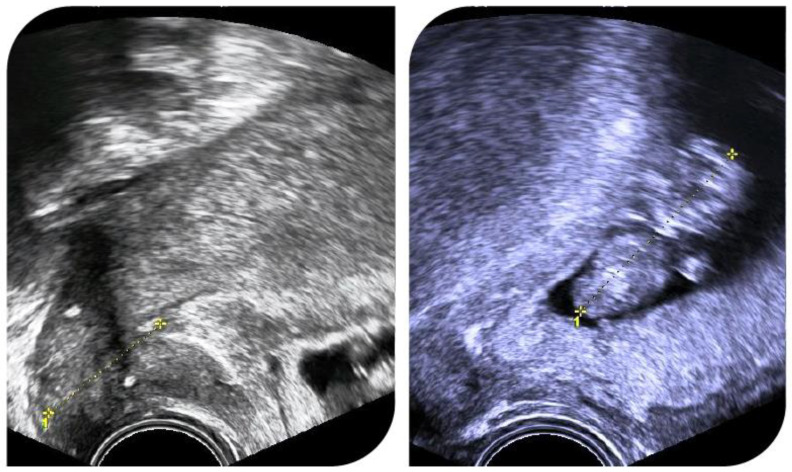
Successful pregnancy after radical abdominal trachelectomy. A cerclage was placed during trachelectomy.

Figure 2 shows ultrasound images of a pregnant patient with cervical length measurements and cerclage position.

## 4. Discussion

In a cohort comprising 79.3% non-“low-risk” tumors, no recurrence following radical abdominal trachelectomy was observed at a median follow-up of more than 5 years. In two out of three cases, neoadjuvant chemotherapy allowed for subsequently successful fertility preservation in patients with 40 mm tumors and previous infiltration of the upper cervical canal. Interestingly, a notable number of patients did not try to conceive despite having opted for fertility-sparing management before; in eight patients who tried to conceive, the live birth rate following radical trachelectomy was 62.5%.

The present findings are in line with those previously reported in the literature. Radical abdominal trachelectomies in adequately selected patients are considered oncologically safe and not inferior compared with radical abdominal hysterectomies [19]. Even though the evidence is limited, previous studies demonstrated that neoadjuvant approaches following abdominal and vaginal trachelectomy are feasible in selected patients [20,21]. Data on fertility outcomes following radical trachelectomy, however, is heterogeneous. Even though several studies have reported successful live births following radical trachelectomy, the number of patients who actually wish to conceive after surgery, pregnancy complications, and live birth rates are difficult to assess due to inherent issues of retrospective analyses on this sensitive topic.

The reproductive outcomes of patients with cervical cancer larger than 2 cm were examined in a systematic review. A total of 443 patients with cervical cancer larger than 2 cm were included. There were eighty pregnancies with 24 miscarriages and 54 live births. This could indicate that in selected individual cases, fertility-preserving therapy may also be possible in tumor stages higher than IB1. However, the oncological outcome should also be investigated in further studies in order to make a general recommendation [22].

Moreover, different cultural aspects may also play a role in deciding on fertility-sparing therapy approaches and reported attempts to conceive after surgery. To date, the literature describes pregnancy rates among patients who wished to conceive around 36–44%, at a considerable risk of preterm birth. According to data by Nezhat et al., pregnancy rates following vaginal trachelectomies could be higher compared with abdominal trachelectomies [19]. The reason for this assumed difference is yet to be elucidated. Of note, most respective evidence is derived from east-Asian patient cohorts [23,24,25]. As data on this topic are extremely limited for central European patients, presently collected data may serve as a starting point for future studies exploring the reasons why only a fraction of patients undergoing a trachelectomy who apparently wish to conceive and if affected patients could profit from better counseling or more multidisciplinary medical support offers (e.g., reproductive endocrinologists and clinical psychologists).

Globally, fertility-sparing trachelectomy for early cervical cancer remains a rarely applied procedure. A recently published survey by Matsuo et al. in the United States described 815 cases of trachelectomies performed in 89 centers over ten years (2001 to 2011). The vast majority of centers included in this survey (76.4%) had a limited surgical volume of just one case per year [26]. Even though the technique of abdominal radical trachelectomy is similar to abdominal radical hysterectomy, it remains a challenging surgical technique, and a certain learning curve has to be considered [27]. A higher surgical volume is associated with reduced perioperative morbidity, and pooling cases of radical trachelectomies in high-volume gyneco-oncological centers is highly recommended [26]. Even though data for Central Europe are limited, our institution’s case numbers, surgical quality indicators, and outcomes are comparable to average tertiary care centers in the United States (>2 cases per year) [26,27,28].

Analogously, selected patients with early-stage endometrial carcinoma can be considered for fertility-preserving therapeutic approaches; similarly, affected patients desiring to preserve fertility should be referred to specialized tertiary care centers in order to optimize the oncological and obstetric outcomes [29,30].

One of the most important future challenges in planning fertility-sparing approaches for early cervical cancer patients will be to balance surgical safety against the risk of morbidity. Given recent promising data on the safety of less radical approaches, accurate patient selection will be paramount [12,13,14]. Even though most herein assessed patients underwent treatment before the safety of simple trachelectomies was established, and around 80% of our cases would not qualify as “low-risk” based on final histopathological results, the present findings highlight the importance of accurate preoperative staging to avoid overtreatment in future.

Incorporating human papillomavirus (HPV) risk stratification algorithms could help to improve the clinical management of patients referred to radical trachelectomy in the future. HPV follow-up tests after trachelectomy could help to facilitate early diagnosis of recurrence, as previously reported among cervical cancer survivors after radiotherapy. Emerging data indicate that the type of HPV strain could be associated with different recurrence risks [31,32].

This study has limitations. As is typical for retrospective analyses, the lack of random patient assignment, patient selection, and potentially incomplete data acquisition limit its clinical applicability. Additionally, given the sample size of existing systematic reviews, the number of patients in our cohort is limited. However, evaluating strictly consecutive patients over a specified timeframe may help alleviate respective biases. The presented data aligns with previous reports and confirms the safety of this procedure from an Austrian perspective, as this series is the first to describe outcomes of radical abdominal trachelectomies in German-speaking countries in a non-”low-risk” cohort with long-term follow-up. In light of emerging data on the safety of more conservative surgical approaches for selected patients with early cervical cancer with low-risk features, e.g., cold-knife conizations or favoring simple trachelectomies over radical trachelectomies, case numbers of performed radical trachelectomies will inevitably decline even in specialized centers. As our department has been the only referral center offering radical abdominal trachelectomies in Austria in the past two decades, and more conservative approaches were usually performed in other local centers, our case management may serve as a role model for the centralization of a rare gyneco-oncologic intervention in future, demonstrating that performing radical trachelectomies remains a safe and effective approach in highly selected patients with early cervical cancer who wish to preserve fertility [12,13].

## 5. Conclusions

Abdominal radical trachelectomy is considered an oncologically safe procedure for early-stage cervical cancer patients who do not meet the “low-risk” criteria and desire fertility preservation. As emerging data support the safety of simple trachelectomies in well-selected patients, achieving the most accurate pretherapeutic staging to balance oncologic safety against potential morbidity will be crucial in the future. With the increasing complexity of accurately selecting patients for specific fertility-sparing procedures, trachelectomies should be exclusively performed at high-volume gynecologic oncology centers, with extensive patient education being essential to clarify obstetric desires and risks before therapy initiation.

## Figures and Tables

**Table 1:** Clinical baseline characteristics of both pretherapeutic clinicopathologic variables and surgical outcomes.

Parameter	N (%), Median (IQR)
Number of patients	29 (100)
Age (years)	31.0 (27.0–36.0)
BMI	21.3 (20.1–22.9)
History of previous pregnancy	3 (10.3)
FIGO stage (2018 system)	
1A2	3 (10.3)
1B1	16 (55.2)
1B2	6 (20.7)
IIC1	4 (13.8)
Histology	
Squamous	24 (82.8)
Adenocarcinoma	5 (17.2)
Grading (G)	
G1	4 (13.8)
G2	17 (58.6)
G3	8 (27.6)
Tumor size (mm)	20 (10.5–26.5)
Stromal invasion ≥10 mm	7 (24.1)
Presence of LVSI	11 (37.9)
Would have fulfilled ConCerv criteria	6 (20.7)
Lymph node staging performed	29 (100)
Systematic node resection	14 (48.3)
Sentinel lymph node resection	15 (51.7)
Width of resected parametria (mm)	30 (15.0–36.3)
Parametrial lymph nodes resected	9 (31.0)
Surgical time (min)	285 (260–305)
Postoperative complications Clavien–Dindo classification	
Grade 1	20 (69.0)
Grade 2	6 (20.7)
Grade 3	3 (10.3)
Follow-up (months)	64.5 (25.5–104.0)

**Table 2:** Obstetric outcomes following radical abdominal trachelectomy.

Parameter		N (%), Median (IQR)
Successful radical abdominal trachelectomies		22 (100)
Women attempting pregnancy	Yes	8 (36.4)
	No	14 (63.6)
Reason for not trying to conceive		
No partner		2 (14.3)
Anxiety about premature birth		1 (7.1)
Anxiety about recurrence of cervical cancer		3 (21.4)
Pregnancy planned in near future		3 (21.4)
No further wish to conceive, no specific reason		5 (20.8)
Clinical pregnancy rate		5 (25.0)
Conception	Assisted reproduction	1 (20.0)
	Spontaneous	4 (80.0)
Cerclage during trachelectomy	Yes	5 (100)
First-trimester miscarriage		1 (20.0)
Second-trimester deliveries		0 (0.0)
Third-trimester deliveries		4 (80.0)
Birth procedure	Cesarean	4 (80.0)
Gestational age		37 + 3 (34 + 6–38 + 3)

## Data Availability

The data supporting the conclusions of this article will be made available by the authors on reasonable request.

## References

[1] Piver M.S., Rutledge F., Smith J.P. (1974). Five classes of extended hysterectomy for women with cervical cancer. Obstet. Gynecol..

[2] Siegel R.L., Giaquinto A.N., Jemal A. (2024). Cancer statistics, 2024. CA Cancer J. Clin..

[3] Laganà A.S., La Rosa V.L., Rapisarda A.M.C., Platania A., Vitale S.G. (2017). Psychological impact of fertility preservation techniques in women with gynaecological cancer. Ecancermedicalscience.

[4] Dargent D., Martin X., Sacchetoni A., Mathevet P. (2000). Laparoscopic vaginal radical trachelectomy: A treatment to preserve the fertility of cervical carcinoma patients. Cancer.

[5] Smith J.R., Boyle D.C., Corless D.J., Ungar L., Lawson A.D., Priore G.D., McCall J.M., Lindsay I., Bridges J.E. (1997). Abdominal radical trachelectomy: A new surgical technique for the conservative management of cervical carcinoma. Br. J. Obstet. Gynaecol..

[6] Tamauchi S., Iyoshi S., Yoshihara M., Yoshida K., Ikeda Y., Shimizu Y., Yokoi A., Niimi K., Yoshikawa N., Kajiyama H. (2024). An update of oncologic and obstetric outcomes of radical trachelectomy for early-stage cervical cancer: The need for further minimally invasive treatment. J. Obstet. Gynaecol. Res..

[7] Kasuga Y., Hasegawa K., Hamuro A., Fukuma Y., Tamai J., Tanaka Y., Ikenoue S., Tanaka M. (2024). Pregnancy outcomes following radical trachelectomy for early-stage cervical cancer: A retrospective observational study in the Kanto area, Japan. Int. J. Gynaecol. Obstet..

[8] Marchiole P., Benchaib M., Buenerd A., Lazlo E., Dargent D., Mathevet P. (2007). Oncological safety of laparoscopic-assisted vaginal radical trachelectomy (LARVT or Dargent’s operation): A comparative study with laparoscopic-assisted vaginal radical hysterectomy (LARVH). Gynecol. Oncol..

[9] Lanowska M., Mangler M., Spek A., Grittner U., Hasenbein K., Chiantera V., Hertel H., Schneider A., Köhler C., Speiser D. (2011). Radical vaginal trachelectomy (RVT) combined with laparoscopic lymphadenectomy: Prospective study of 225 patients with early-stage cervical cancer. Int. J. Gynecol. Cancer..

[10] Xu L., Sun F.Q., Wang Z.H. (2011). Radical trachelectomy versus radical hysterectomy for the treatment of early cervical cancer: A systematic review. Acta Obstet. Gynecol. Scand..

[11] Prodromidou A., Iavazzo C., Fotiou A., Psomiadou V., Douligeris A., Vorgias G., Kalinoglou N. (2019). Short- and long term outcomes after abdominal radical trachelectomy versus radical hysterectomy for early stage cervical cancer: A systematic review of the literature and meta-analysis. Arch. Gynecol. Obstet..

[12] Schmeler K.M., Pareja R., Blanco A.L., Fregnani J.H., Lopes A., Perrotta M., Tsunoda A.T., Cantú-De-León D.F., Ramondetta L.M., Manchana T. (2021). ConCerv: A prospective trial of conservative surgery for low-risk early-stage cervical cancer. Int. J. Gynecol. Cancer..

[13] Plante M., Renaud M.C., Sebastianelli A., Gregoire J. (2020). Simple vaginal trachelectomy in women with early-stage low-risk cervical cancer who wish to preserve fertility: The new standard of care?. Int. J. Gynecol. Cancer..

[14] Plante M., Kwon J.S., Ferguson S., Samouëlian V., Ferron G., Maulard A., de Kroon C., Van Driel W., Tidy J., Williamson K. (2024). Simple versus Radical Hysterectomy in Women with Low-Risk Cervical Cancer. N. Engl. J. Med..

[15] Speiser D., Malik S., Lanowska M., Bartens A., Blohmer J.U., Mangler M. (2017). Follow-up after radical vaginal trachelectomy (RVT): Patients’ problems and physicians’ difficulties. Arch. Gynecol. Obstet..

[16] Querleu D., Morrow C.P. (2008). Classification of radical hysterectomy. Lancet Oncol..

[17] Querleu D., Cibula D., Abu-Rustum N.R. (2017). 2017 Update on the Querleu-Morrow Classification of Radical Hysterectomy. Ann Surg Oncol..

[18] Singh R.B., Chander S., Mohanti B.K., Pathy S., Kumar S., Bhatla N., Thulkar S., Vishnubhatla S., Kumar L. (2013). Neoadjuvant chemotherapy with weekly paclitaxel and carboplatin followed by chemoradiation in locally advanced cervical carcinoma: A pilot study. Gynecol. Oncol..

[19] Nezhat C., Roman R.A., Rambhatla A., Nezhat F. (2020). Reproductive and oncologic outcomes after fertility-sparing surgery for early stage cervical cancer: A systematic review. Fertil. Steril..

[20] Pareja R., Rendón G.J., Vasquez M., Echeverri L., Sanz-Lomana C.M., Ramirez P.T. (2015). Immediate radical trachelectomy versus neoadjuvant chemotherapy followed by conservative surgery for patients with stage IB1 cervical cancer with tumors 2cm or larger: A literature review and analysis of oncological and obstetrical outcomes. Gynecol. Oncol..

[21] Ferraioli D., Mathevet P., Chopin N., Beurrier F., Tigaud J.D. (2017). Neoadjuvant chemotherapy followed by vaginal radical trachelectomy to preserve the fertility in young patients with large cervical carcinoma. Gynecol. Oncol..

[22] D’Amato A., Riemma G., Agrifloglio V., Chiantera V., Laganà A.S., Mikus M., Dellino M., Maglione A., Faioli R., Giannini A. (2024). Reproductive Outcomes in Young Women with Early-Stage Cervical Cancer Greater Than 2 cm Undergoing Fertility-Sparing Treatment: A Systematic Review. Medicina.

[23] Nishio H., Fujii T., Sugiyama J., Kuji N., Tanaka M., Hamatani T., Miyakoshi K., Minegishi K., Tsuda H., Iwata T. (2013). Reproductive and obstetric outcomes after radical abdominal trachelectomy for early-stage cervical cancer in a series of 31 pregnancies. Hum. Reprod..

[24] Kasuga Y., Nishio H., Miyakoshi K., Sato S., Sugiyama J., Matsumoto T., Tanaka K., Ochiai D., Minegishi K., Hamatani T. (2016). Pregnancy Outcomes after Abdominal Radical Trachelectomy for Early-Stage Cervical Cancer: A 13-Year Experience in a Single Tertiary-Care Center. Int. J. Gynecol. Cancer..

[25] Terzic M., Makhadiyeva D., Bila J., Andjic M., Dotlic J., Aimagambetova G., Sarria-Santamera A., Laganà A.S., Chiantera V., Vukovic I. (2023). Reproductive and Obstetric Outcomes after Fertility-Sparing Treatments for Cervical Cancer: Current Approach and Future Directions. J. Clin. Med..

[26] Matsuo K., Matsuzaki S., Mandelbaum R.S., Matsushima K., Klar M., Grubbs B.H., Roman L.D., Wright J.D. (2020). Association between hospital surgical volume and perioperative outcomes of fertility-sparing trachelectomy for cervical cancer: A national study in the United States. Gynecol. Oncol..

[27] Pareja R., Rendón G.J., Sanz-Lomana C.M., Monzón O., Ramirez P.T. (2013). Surgical, oncological, and obstetrical outcomes after abdominal radical trachelectomy—A systematic literature review. Gynecol. Oncol..

[28] Smith E.S., Moon A.S., O’Hanlon R., Leitao M., Sonoda Y., Abu-Rustum N.R., Mueller J. (2020). Radical Trachelectomy for the Treatment of Early-Stage Cervical Cancer: A Systematic Review. Obstet. Gynecol..

[29] Cavaliere A.F., Perelli F., Zaami S., D’Indinosante M., Turrini I., Giusti M., Gullo G., Vizzielli G., Mattei A., Scambia G. (2021). Fertility Sparing Treatments in Endometrial Cancer Patients: The Potential Role of the New Molecular Classification. Int. J. Mol. Sci..

[30] Etrusco A., Laganà A.S., Chiantera V., Mikuš M., Muhammad Arsalan H., d’Amati A., Vitagliano A., Cicinelli E., Favilli A., D’Amato A. (2024). Reproductive and Oncologic Outcomes in Young Women with Stage IA and Grade 2 Endometrial Carcinoma Undergoing Fertility-Sparing Treatment: A Systematic Review. Biomolecules.

[31] Despot A., Fureš R., Despot A.M., Mikuš M., Zlopaša G., D’Amato A., Chiantera V., Serra P., Etrusco A., Laganà A.S. (2023). Reactive oxygen species within the vaginal space: An additional promoter of cervical intraepithelial neoplasia and uterine cervical cancer development?. Open Med..

[32] Sabeena S., Kuriakose S., Damodaran B., Ravishankar N., Arunkumar G. (2020). Human papillomavirus (HPV) DNA detection in uterine cervix cancer after radiation indicating recurrence: A systematic review and meta-analysis. J. Gynecol. Oncol..

